# Inhibition of immune checkpoints PD-1, CTLA-4, and IDO1 coordinately induces immune-mediated liver injury in mice

**DOI:** 10.1371/journal.pone.0217276

**Published:** 2019-05-21

**Authors:** Timothy Affolter, Heather P. Llewellyn, Derek W. Bartlett, Qing Zong, Shuhua Xia, Vince Torti, Changhua Ji

**Affiliations:** 1 Global Pathology, Pfizer Drug Safety Research and Development, La Jolla, California, United States of America; 2 Medicine Design, Pfizer Worldwide Research and Development, La Jolla, California, United States of America; 3 Biomarkers, Pfizer Drug Safety Research and Development, La Jolla, California, United States of America; 4 Investigative Toxicology, Drug Safety Research and Development, Groton, Connecticut, United States of America; 5 General Toxicology, Drug Safety Research and Development La Jolla, California, United States of America; National Institutes of Health, UNITED STATES

## Abstract

Cancer cells harness immune checkpoints such as cytotoxic T-lymphocyte-associated protein 4 (CTLA-4), programmed cell death protein 1 (PD-1) and indoleamine 2,3-dioxygenase 1 (IDO1) to evade immune control. Checkpoint inhibitors have demonstrated durable anti-tumor efficacy in human and preclinical models. Liver toxicity is one of the common immune-related adverse events associated with checkpoint inhibitors (CPIs) and its frequency and severity often increase significantly during CPI combination therapies. We aim to develop a mouse model to elucidate the immune mechanisms of CPI-associated liver toxicity. Co-administration of CTLA-4 blocking antibody, 9D9, and/or an IDO1 inhibitor, epacadostat in wild-type and *PD-1*^*-/-*^ mice (to simulate the effect of PD1 blockade) synergistically induced liver injury and immune cell infiltration. Infiltrated cells were primarily composed of CD8+ T cells and positively associated with hepatocyte necrosis. Strikingly, sites of hepatocyte necrosis were frequently surrounded by clusters of mononuclear immune cells. CPI treatments resulted in increased expression of genes associated with hepatocyte cell death, leukocyte migration and T cell activation in the liver. In conclusion, blockade of immune checkpoints PD-1, CTLA-4, and IDO1 act synergistically to enhance T cell infiltration and activity in the liver, leading to hepatocyte death.

## Introduction

Inhibition of CTLA-4 (cytotoxic T-lymphocyte-associated protein 4), PD-1 (programmed cell death 1) and IDO1 (indoleamine 2,3-dioxygenase 1) has demonstrated antitumor efficacy in preclinical models and humans across several types of cancers [1–10]. In general, immune checkpoint inhibitors (CPIs) block T cell inhibition and promote tumor cell killing [11, 12]. However, as many of these pathways have been shown to also be important in promoting liver immune tolerance, liver immune-related adverse events are frequently observed in cancer patients treated with CPIs. This immune-mediated liver injury induced by CPIs is considered a novel type of hepatotoxicity and is distinct from other types of drug induced liver injury.

CTLA-4 is primarily expressed on CD4+ and CD8+ T cells in humans and mice [13] during the priming phase of effector T cell activation and is a co-inhibitory signal upon binding to CD80 or CD86 on antigen presenting cells. Genetic deletion of CTLA-4 in mice leads to generalized hyper-lymphoproliferative disorder and multi-tissue (including the liver) accumulation of self-reactive T cells [14, 15], suggestive of a break in immune tolerance. Similar immunological changes and disease presentations were also observed in patients treated with CTLA-4 blocking antibodies [16], indicating that CTLA-4 has similar functions in mouse and human.

PD-1 is an important mediator of the induction and maintenance of immunologic tolerance. PD-1 is expressed on activated T cells, B cells and myeloid cells. In T cells, upregulation of PD-1 negatively regulates T cell receptor signaling upon binding to one of its ligands, PD-L1 or PD-L2 [17]. In the murine liver, PD-L1 is expressed on hepatocytes, hepatic stellate cells, liver sinusoidal endothelial cells and Kupffer cells, and PD-L2 is expressed on liver sinusoidal endothelial cells, Kupffer cells, and intrahepatic leukocytes. Engagement of PD-1 on regulatory T cells (Tregs) may also contribute to immune tolerance in the liver [13].

The immune modulator IDO1 is an intracellular enzyme that degrades L-tryptophan along the L-kynurenine pathway. Decreased L-tryptophan can inhibit T cell activation and proliferation, and L-kynurenine promotes Treg activity. IDO1 can be induced in the liver by inflammatory stimuli [18]. Hepatic stellate cells can induce tolerogenic dendritic cells by inducing IDO1 expression [19]. Furthermore, liver injury stimuli can promote inflammation in IDO1-/- mice [18, 20].

Ipilimumab, a CTLA-4 blocking antibody, was the first FDA approved CPI [21]. The frequency and severity of liver toxicity was markedly increased when ipilimumab was used in combination with IDO1 inhibitor epacadostat at 300 mg twice a day (BID) [22]. The combination of ipilimumab with nivolumab, a PD-1 blocking antibody, also increased the frequency of grade 3/4 liver toxicity by more than 5-fold [2]. IDO1 inhibitors are currently in several clinical trials largely in combination with anti-PD1 or anti-PDL1 antagonists [1]. A clinical trial (NCT03347123) is testing the combination of anti-CTLA-4, anti-PD-1 and epacadostat in advanced cancer. CTLA-4 blocking antibody induces liver lymphocyte accumulation which is exacerbated with the addition of anti-PD-1 in mice [23]. The mechanisms of enhanced hepatotoxicity when combining CPIs are yet to be elucidated.

We hypothesize that the simultaneous inhibition of PD-1, CTLA-4, and IDO1 potentiates liver injury via T cell expansion and breaks immune tolerance in the liver microenvironment. Restricted use of liver biopsies limited the study of mechanisms of CPI therapy related liver toxicities. Here we demonstrate that administration of an anti-CTLA-4 antibody and an IDO1 inhibitor, in combination, to *PD-1*^*-/-*^ mice can recapitulate the clinical enhanced liver toxicity associated with the combination of these CPIs. Moreover, the liver injuries in our mouse model are characterized by hepatocyte necrosis surrounded by clusters of lymphocytes. And these histological presentations are similar to what have been reported in patients receiving CPI therapies.

## Materials and methods

### Mice

Female wild-type C57BL/6N (Charles River Laboratories) mice, 13–15 weeks old, 20-25g; and female *PD-1*^*-/-*^ mice (B6.Cg-Pdcd1^tm1.1Shr^) on a C57BL/6N background (Taconic Biosciences), 24–30 weeks old, 22-32g, were used in this experiment. The *PD-1*^*-/-*^ mice have been previously described [24]. The study was conducted in accordance with the current guidelines for animal welfare (National Research Council Guide for the Care and Use of Laboratory Animals, 2011; Animal Welfare Act, 1966, as amended in 1970, 1976, 1985, and 1990, and the Animal Welfare Act implementing regulations in Title 9, Code of Federal Regulations, Chapter 1, Subchapter A, Parts 1–3). The procedures used in this study have been reviewed and approved by Pfizer’s Institutional Animal Care and Use Committee. Animals were acclimated to the laboratory environment for at least three days prior to dosing, co-housed to reduce microbiome effects, and randomly assigned to experimental groups via cage assignment. The investigators were not blinded to each experimental group during dosing, data collection or analysis. Animals were housed under routine 12-hour light cycle-controlled conditions with standard corncob bedding (Shepherd Specialty Papers Alpha-Dri/bed-o-Cob). Animals were on standard chow (Rodent Diet 5L0D, PicoLab) and had free access to food and water without any fasting.

### Vehicle(s) and experimental test articles

The IDO1 inhibitor epacadostat (Chemscene, Monmouth Junction, NJ) was administered twice daily (6 hours between each dose) as an oral gavage formulation with 0.5% HPMC/0.25% Tween 20 in a water base at 100, 300 or 600 mg/kg/dose. The anti-CTLA-4 antibody 9D9 (BioXcell) was administered intravenously at 300 ug/dose in phosphate buffered saline (PBS) solution.

Wild-type mice (6 mice per group) were administered 9D9 (anti-CTLA-4) or PBS vehicle control on days 1, 4, 11, 18, and 25 of a 32-day study. Epacadostat (300 mg/kg/dose) or vehicle control was administered twice daily, 6 hours between doses, on days 4–31. The mice were humanely euthanized by exsanguination while under isoflurane gas anesthesia on day 32.

To assess the time course of effects, the study using PD1-/- mice contained two cohorts. One cohort of mice (6 per group) was administered anti-CTLA-4 (9D9) on days 1, 4, and 11 and epacadostat (300 or 600 mg/kg/dose BID) or vehicle control on days 4–17. A second cohort of 6 mice per group was dosed with anti-CTLA-4 or PBS control on days 1, 4, 11, 18, 25, 32, and 39; and epacadostat (600 mg/kg/dose BID) or vehicle control twice daily on days 4–45. An additional cohort of 12 mice per group was dosed with anti-CTLA-4 or PBS control on days 1, 4, 11, 18; and epacadostat (600 mg/kg/dose BID) or vehicle control twice daily on days 4–22. Humane euthanasia and necropsy were performed on days 18, 22 and 46, for the first, second and additional cohort, respectively.

### Histopathology and immunohistochemistry

Standard hematoxylin and eosin-stained liver sections were prepared for each animal for microscopic evaluation following fixation in 10% neutral-buffered formalin. Lesions were subsequently classified by a board-certified pathologist (author TA) according to a semi-quantitative scoring scheme. Scoring schema for mixed cell infiltrates, immunohistochemistry for lymphocyte markers and necrosis was 0 = baseline set at 1.5x the amount seen in highest control for each of the regions scored (portal, mid-zonal/centrilobular), 1 = minimal increase from baseline, 2 = mild increase from baseline, 3 = moderate increase from baseline, and 4 = marked increase from baseline. Mixed cell infiltrates (or, ‘infiltrates’) and single cell necrosis were scored from H & E sections. Mixed cell infiltrates refer to periportal clusters of predominantly mononuclear immune cells. Inflammation/necrosis refers to individual or small clusters of necrotic hepatocytes encircled by mononuclear cells primarily within mid-zonal and centrilobular regions. For liver, four sections from each animal were evaluated (from comparable areas within the left and right medial and left and right lateral lobes). For the immunohistochemical marker evaluations, separate scores were given for two subpopulations of infiltrates in the liver, because the subpopulations have different pathobiologic implications. The first category was assigned to mononuclear cell populations restricted to periportal zones of the liver, which generally exemplify an exacerbation or expansion of a pre-existing population of resident mononuclear leukocytes, and the second category was applied to variable infiltrates primarily identified in mid-zonal and centrilobular zones frequently associated with necrotic foci.

Immunohistochemistry for CD4, CD8, and Foxp3 was applied to slides of liver with each single slide containing four sections of liver from one animal (four lobes). Anti-CD4 (4SM9, 1:400), anti-CD8α (4SM15, 1:1200) and Foxp3 (Fjk16s, 1:200) were purchased from ThermoFisher (Waltham, MA, USA). All primary antibodies were incubated for 30 minutes. Antigen retrieval for all markers was performed using Bond HIER epitope retrieval solution (Leica Biosystems) for 20 minutes. Detection was performed with the Bond Polymer Refine kit (Leica Biosystems) on a Leica Bond III automated stainer. Immunoreactivity scoring was performed using a similar semi-quantitative scale as used for the hematoxylin and eosin-stained sections summarized above. Each individual score for each animal represents an average of each of the four sections of liver on a single slide representing one animal.

### Epacadostat pharmacokinetics and pharmacodynamics

Wild-type and *PD-1*^*-/-*^ C57BL/6 mice were administered epacadostat (100, 300, and 600 mg/kg/dose) in two doses six hours apart using an oral gavage formulation with 0.5% HPMC/0.25% Tween 20 in a water base. Plasma samples (n = 3 mice per timepoint) were collected at 0 h (pre-first dose), 1 h, 2 h, 4 h, 6 h (pre-second dose), 7 h, and 24 h. The concentrations of epacadostat and kynurenine, a pharmacodynamic marker, in the plasma samples were determined by LC-MS/MS.

### Flow cytometry

The middle liver lobe was minced in cold Roswell Park Memorial Institute medium (RPMI-1640, Gibco) and transferred to a mixture of 20 mg/mL Collagenase I (source) and 2.0 mg/mL Hyaluronidase in RPMI. Samples were incubated for 20 minutes at 37C with intermittent mixing. RPMI with 5% fetal bovine serum (Gibco) was added to inhibit the digestion and the single cell suspension was filtered through a 70 μM mesh strainer. Suspended cells were surface labeled with the following fluorescent antibodies from BD bioscience: APC-R700 CD45 (30-F11), FITC CD3 (17A2), PE-Cy7 CD4 (RM4-5), BV605 CD8 (53–6.7), V450 CD49b (DX5), PE F4/80 (T45-2342), BV510 CD11b (M1/70), and APC Gr1 (RB6-8C5). Intracellular staining for Foxp3 (FJK-16s) and Ki67 (SolA15) was performed with the eBioscience Foxp3 / Transcription Factor Staining Buffer Set following the manufacturer’s instructions. Cell data were acquired on a BD FACSCanto and analyzed with Flowjo, LLC software. Each data point represents one animal. One WT mouse was an outlier and was excluded from the flow cytometric analysis of leukocytes and T cell populations.

### GLDH measurement

GLDH was measured as part of a serum chemistry panel which included other measurements of liver injury such as alanine aminotransferase, aspartate aminotransferase, alkaline phosphatase, albumin and bilirubin. The serum chemistry panel was measured by an Advia 1200 Chemistry analyzer (Siemens Healthcare Diagnostics). Each measurement represents one individual animal.

### RNA isolation

Mice were sacrificed from vehicle control, 9D9, epacadostat and cohorts (9D9+epacadostat) at day 18 after treatment. Livers were collected at necropsy, embedded in optimal cutting medium (OCT) and stored at -80°C. Prior to cutting, frozen tissue sections were warmed to − 20 °C, cut into 10 μm sections using Leica CM3050 (Leica, IL); 4 sections from each tissue were subjected to RNA isolation.

RNA extraction was performed using Qiagen RNeasy Lipid Tissue mini Kit (Qiagen, CA); RNA concentrations were determined by Qubit fluorometer (Thermo Fisher, NJ) and the RNA quality was determined by measuring the RNA integrity number (RIN) using Agilent TapeStation 4200 (Agilent, CA).

### Library preparation and sequencing

50–100 ng/sample RNA from each animal was used as input for cDNA synthesis and amplification with the SMARTer Ultra Low Input RNA kit (Clontech, CA) following manufacturer recommendations with 13 cycles of PCR amplification. cDNA quantity and quality were evaluated with a Qubit fluorometer and TapeStation 4200, respectively.

100pg of amplified cDNA was used to generate DNA libraries using Illumina Nextera XT DNA Library Kit (Illumina, CA). Twelve library amplification cycles were applied per manufacturer’s recommendation. Library quantity and quality were evaluated as described above for the cDNA. Illumina paired-end 151 bp sequencing was performed on a Next-seq 500 apparatus (Illumina, USA).

### RNA-Seq data analysis

Raw reads were aligned to the mouse genome using BaseSpace (Illumina, CA) software RNA Express. Briefly, trimmed reads were mapped to the reference genome using Star 3.0; the expression level was normalized by the number of fragments per kilobase of exon per million mapped reads (FPKM) and differentially expressed genes (DEGs) were detected using DESeq2 with a raw *p* < 0.05 or adjusted p value *q* < 0.05. All DEGs were subjected to Ingenuity Pathway Analysis (IPA, Qiagen, CA). The pathway analysis was limited by species to mouse and only liver organ system and immune cells were used as the knowledge base.

### Statistics

Sample size was determined from previous experiments to achieve statistical significance. Each experiment was performed once. An unpaired, two-tailed t-test was performed to determine significance between two groups. A one-way ANOVA with Tukey’s correction for multiple comparisons was used for comparing more than two groups. The variance was similar between groups when using the t-test or one-way ANOVA. A Kruskal-Wallis test with Dunn’s multiple comparisons test was performed when comparing more than two groups for the histologic data. Statistics were performed with GraphPad Prism 7 (GraphPad Software, La Jolla CA USA). For the association analysis, Goodman and Kruskal’s gamma statistics were used to measure the strength of the association between necrosis and histologic scores defined as ordinal data. The gamma value is reported to measure the strength of the association. Correlation analysis was performed with JMP 12.0.1 software. To determine the relationship between the flow cytometry results and histologic necrosis, a linear regression was performed with GraphPad Prism 7. All data is reported as mean +/- SEM unless noted elsewhere and met the assumptions of each statistical test.

All datasets on which the conclusions of the report rely are available on request.

## Results

### PD-1 deficiency leads to increased T cell infiltration in the liver

**Fig 1:**
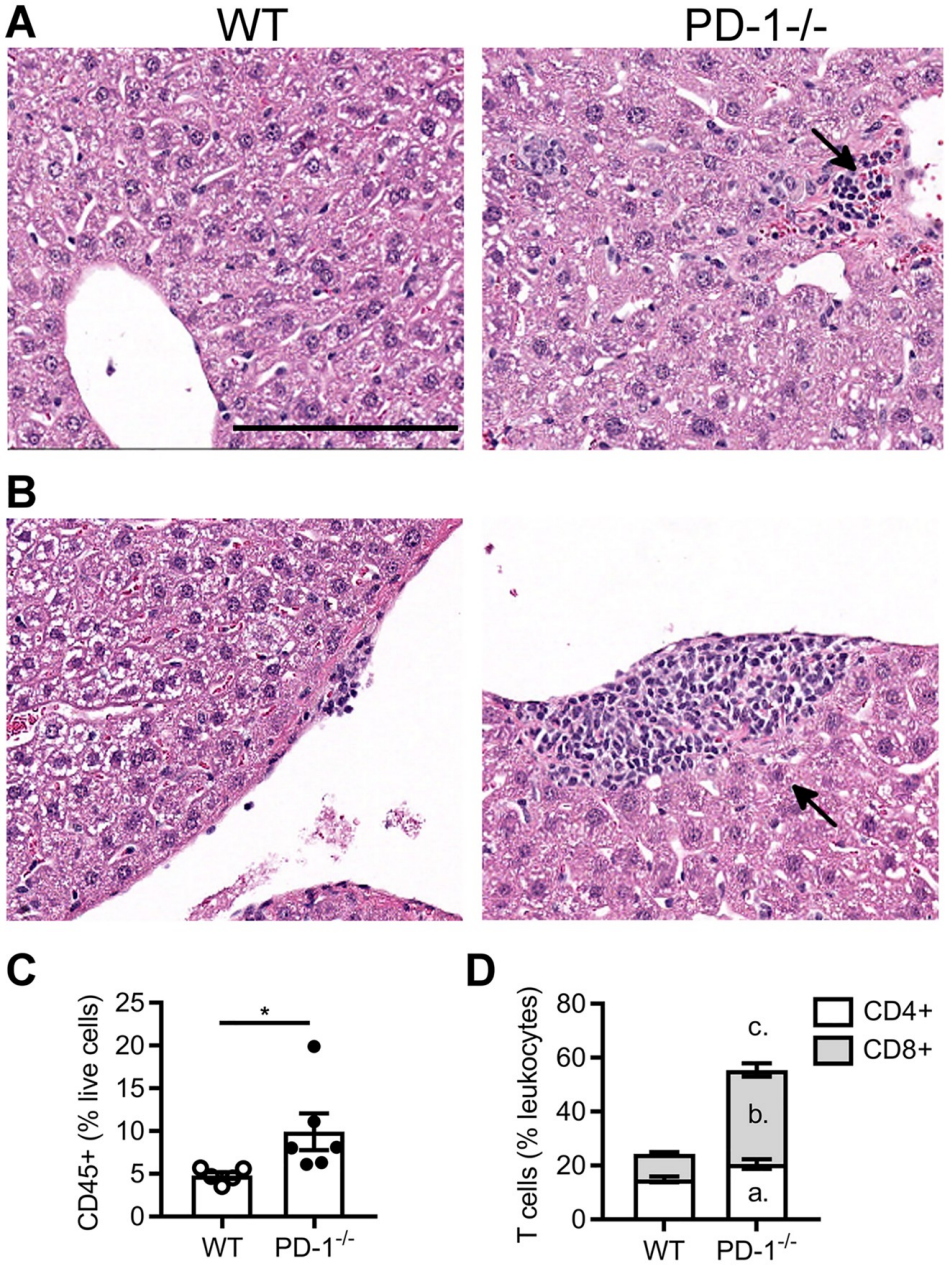
*PD-1*^*-/-*^ mice have increased T cell liver inflammation. Representative H&E images of C57BL/6N (WT) and *PD-1*^*-/-*^ liver in the centrilobular (A) and periportal (B) regions. Quantification of liver (C) CD45+ cells and (D) T cells subsets by flow cytometry. Arrows indicate regions of immune cell accumulation. Scale bar = 150 μm. All data are reported as mean ± SEM. *P<0.05, ^a^P < 0.05 CD4 WT vs *PD-1*^*-/-*^, ^b^P < 0.0001 CD8 WT vs *PD-1*^*-/-*^, ^c^P < 0.0001 total T cells WT vs *PD-1*^*-/-*^, unpaired t test, n = 5 (WT) and 6 (*PD-1*^*-/-*^).

*PD-1*^*-/-*^ mice were used as a model to investigate the potential role of the PD-1 pathway in CPI induced liver injury. Mononuclear cell accumulation was markedly increased in the centrilobular (Fig 1A) and periportal (Fig 1B) regions in livers of *PD-1*^*-/-*^ mice in comparison with compared to C57BL/6N wild-type mice. Flow cytometry analysis of the immune cells in the liver revealed that the percentage of CD45+ immune cells in the total liver cells was higher in the *PD-1*^*-/-*^ mice than in wild-type mice (Fig 1C), predominantly due to greater numbers of CD8+ T cells (Fig 1D). This finding is consistent with previous reports of age-matched mice [25]. Despite increased T cells in the liver, there was no liver injury found in the *PD-1*^*-/-*^ mice, as demonstrated by the lack of hepatocyte inflammation/necrosis (Fig 1A and 1B) or increased serum GLDH compared to wild-type mice.

### Blockade of IDO1 and/or CTLA-4 pathway induces liver injury

**Fig 2:**
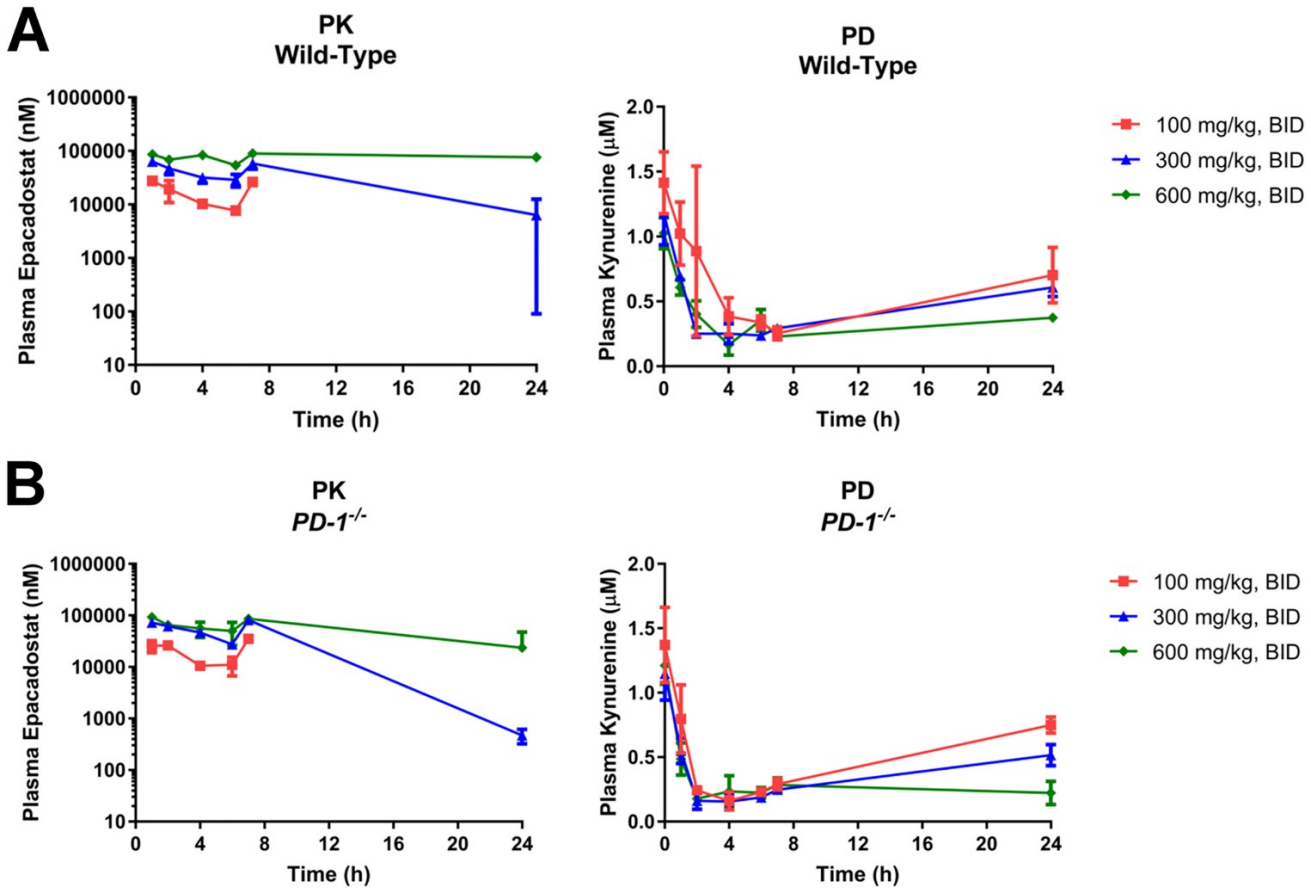
Epacadostat pharmacology. Pharmacokinetics (PK)/pharmacodynamics (PD) in (A) wild-type and (B) *PD-1*^*-/-*^ mice. Mice were administered epacadostat at doses of 100, 300, and 600 mg/kg/dose by oral gavage at t = 0 h and t = 6 h, and total plasma concentrations of epacadostat and kynurenine were measured by LC-MS/MS. Epacadostat concentrations were below the limit of quantification at 24 h for the 100 mg/kg BID dose. All values are reported as mean ± SD. n = 3 (WT) and 3 (*PD-1*^*-/-*^). PD: pharmacodynamics, PK: pharmacokinetics, BID: bis in die (twice daily).

To determine if blockade of IDO1 or CTLA-4 causes liver injury, epacadostat, a small molecule IDO1 inhibitor, and 9D9, a murine CTLA-4 blocking antibody, were administered to wild-type C57BL/6 mice, alone or in combination. 9D9 is a high affinity anti-mouse CTLA-4 monoclonal antibody that showed potent antitumor effects in mouse models. The doses used in the current study were similar to previously reported doses used in various mouse models [26, 27]. To ensure sufficient exposure was achieved to completely block CTLA-4, the 9D9 serum level was determined from blood collected at necropsy. The serum concentrations of 9D9 in all mice were similar and greater than 50 μg/ml, a concentration expected to saturate the CTLA-4 target *in vivo* [26]. For epacadostat, a clinical dose of 300 mg, twice a day (BID), showed high grade liver toxicities in patients when combined with ipilimumab [22]. This dose level was selected as the recommended phase II monotherapy dose to achieve exposures that maintain >90% inhibition of IDO1 activity based on an *ex vivo* stimulated whole blood assay for kynurenine production, with the 90% inhibitory concentration being observed at plasma concentrations greater than 500 nM [28]. Moreover, patients treated with >300 mg BID of epacadostat had ~50% lower plasma kynurenine levels on day 15 of treatment relative to baseline data. Epacadostat has been shown to be less active in mice than in humans [29]. To define the dose levels in the mouse studies that achieve IDO1 inhibition activity similar to that targeted in the human clinical studies, we performed a mouse pharmacokinetics/pharmacodynamics study evaluating the plasma concentrations of epacadostat and kynurenine following oral dosing of epacadostat in wild-type and *PD-1*^*-/-*^ mice. As show in Fig 2, all doses of epacadostat resulted in exposures that exceeded the reported *in vivo* IC50 for epacadostat in human patients (~170 nM on day 1 and ~70 nM on day 15) [30], while doses above 300 mg/kg BID maintained >IC90 levels throughout the 24-hour period in both strains of mice. The observed reductions in plasma kynurenine were consistent with the inhibition of IDO1 activity expected at these epacadostat exposures [29, 31]. All doses tested in the mice were well tolerated, and there were no treatment-related body weight changes or other indicators of distress. Therefore, 300 mg/kg BID or 600 mg/kg BID of epacadostat were used in this mouse model to ensure a sufficient pharmacodynamic effect on IDO1 inhibition.

**Fig 3:**
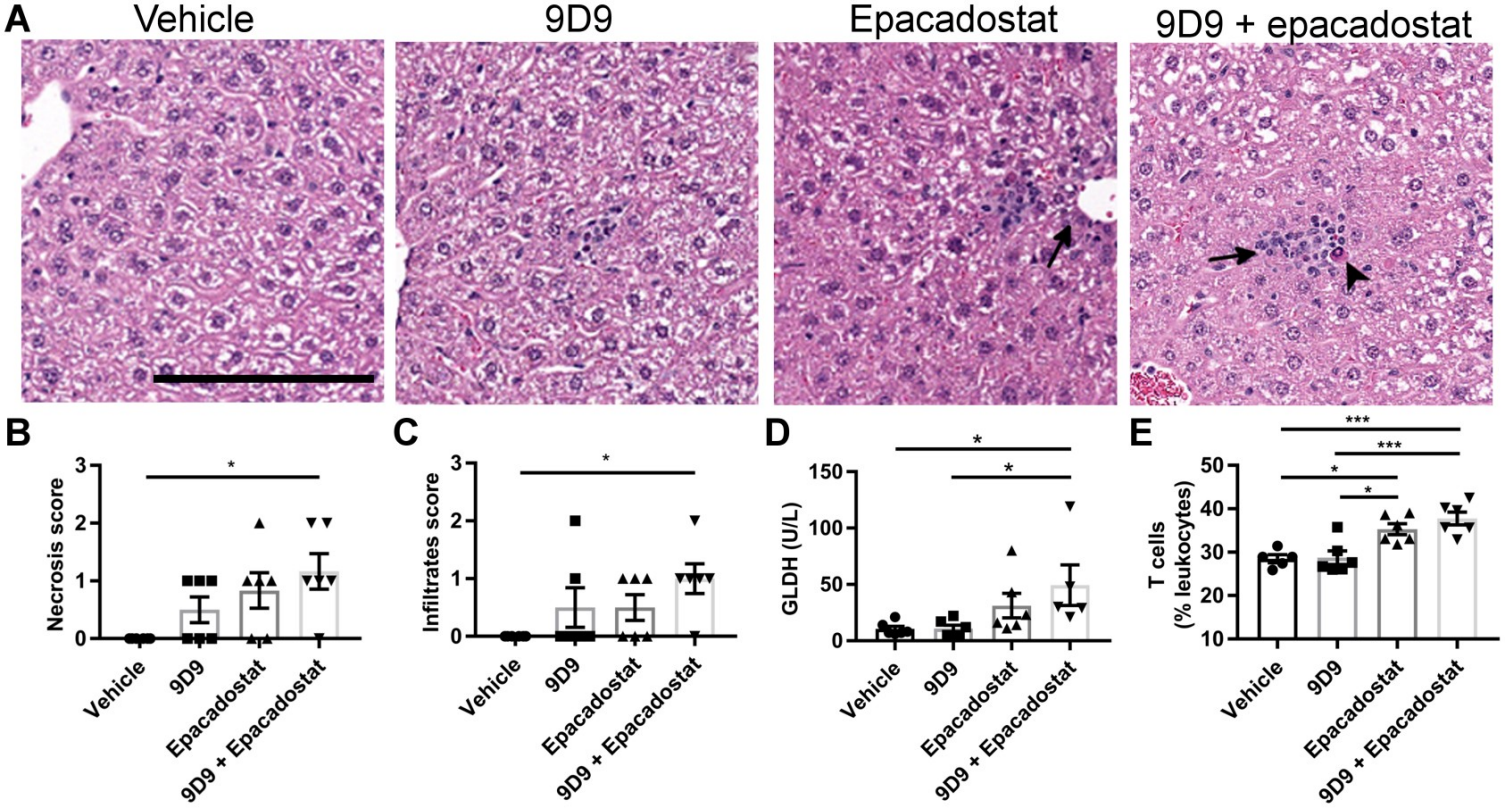
Blockade of IDO1 and CTLA-4 pathways induces liver injury in WT mice. Mice were treated with 300 mg/kg BID epacadostat and/or 9D9 for 4 weeks (see methods). (A) Representative H&E images and quantification of liver (B) single cell necrosis and (C) periportal infiltration. (D) GLDH. (E) Quantification of T cells by flow cytometry. Necrosis, infiltration and GLDH data were analyzed with a Kruskal-Wallis test and Dunn’s multiple comparisons test. Flow cytometry data was analyzed with an ordinary one-way ANOVA with Tukey’s multiple comparisons test. Arrows indicate immune cell accumulation. Arrowheads indicate a necrotic cell. Scale bar = 150 μm. All data are reported as mean ± SEM *P<0.05, ***P<0.001, n = 6 per group. GLDH: glutamate dehydrogenase.

Increases in mononuclear cells in periportal and other regions of the liver (Fig 3A and 3C) as well as appreciable increases in single cell necrosis (Fig 3B) were observed in the single agent treatment groups and were seen with greater severity in the combination group. To assess hepatocyte damage, ALT, AST, and glutamate dehydrogenase (GLDH) were used as biomarkers. However, in this CPI mouse liver injury model, GLDH showed the most consistent and sensitive association with histopathologic necrotic/degenerative changes. As seen in this study, histopathologic characterizations of immune-mediated toxicities may commence as a low-grade, ongoing process of degeneration/necrosis and regeneration of a more insidious nature than that frequently seen with primary hepatocellular toxicities. As GLDH tends to be a more sensitive marker for these types of processes [32] and correlated more tightly with histopathologic findings, we focused on GLDH as the primary liver injury biomarker in the current study. The combination of anti-CTLA-4 and epacadostat significantly increased GLDH levels above the vehicle control and anti-CTLA-4 groups (Fig 3D). Liver immune cells were analyzed by flow cytometry for total immune cells (CD45+) and T cells (CD3+). There were no significant changes of the total CD45+ immune cells after treatment with 9D9, epacadostat, or both. However, there were more T cells in the livers from mice administered epacadostat alone or in combination with 9D9 (Fig 3E).

### Blockade of IDO1 and/or CTLA-4 pathway in *PD-1*^*-/-*^ mice leads to enhanced liver damage

**Fig 4:**
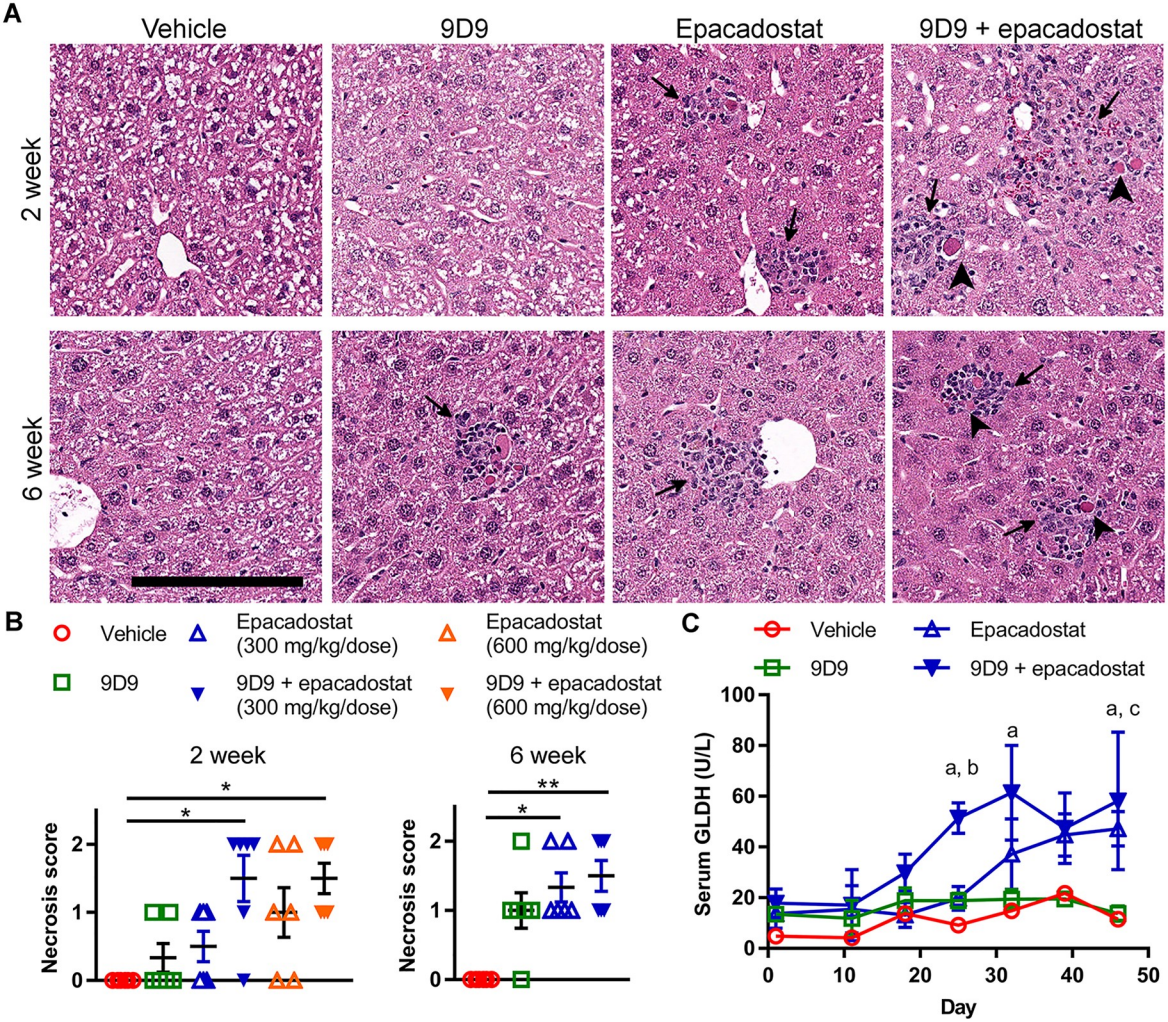
IDO1 and CTLA-4 blockade induces liver injury in *PD-1*^*-/-*^ mice. *PD-1*^*-/-*^ mice were treated with 9D9, epacadostat (A, C panels are 300 mg/kg BID only, B includes 300 and 600 mg/kg BID), or in combination for 2 or 6 weeks. (A) Representative H&E images and (B) quantification of single cell necrosis. (C) GLDH levels through 6 weeks. Arrows indicate immune cell accumulation. Arrowheads indicate a necrotic cell. Scale bar = 150 μm. All data (n = 6 per group) are reported as mean ± SEM. Necrosis data was analyzed with a Kruskal-Wallis test and Dunn’s multiple comparisons test. GLDH data was analyzed with a repeated measures two-way ANOVA and Tukey’s multiple comparisons test. ^a^P < 0.05 9D9 + epacadostat vs vehicle, 9D9, ^b^P < 0.05 9D9 + epacadostat vs epacadostat, ^c^P < 0.05 epacadostat vs vehicle, 9D9.

To evaluate the combinational effect of blocking the three checkpoints CTLA-4, PD-1 and IDO1, we used *PD-1*^*-/-*^ mice instead of an antibody to inhibit PD-1 pathway. As described above, although increased T cell numbers were observed in the livers of *PD-1*^*-/-*^ mice, no histologic evidence of liver injury was identified in these mice. Thus, *PD-1*^*-/-*^ mice were concluded to be suitable for testing the combinational effects of blocking PD-1 and CTLA-4 or PD-1 and IDO1, or all these three checkpoint pathways, on the induction of liver injury. *PD-1*^*-/-*^ mice were administered epacadostat or 9D9 alone or in combination as described in the methods. Epacadostat was administered at 300 and 600 mg/kg/dose BID. After 2 weeks of treatment, single cell necrosis was found in periportal and other regions of the liver in the single-agent and combination treatment groups (Fig 4A and 4B). Minimal increases in inflammation/necrosis were seen in the group treated with anti-CTLA-4 alone (2/6) and in the group treated with epacadostat 300 mg/kg/dose BID alone (3/6). Minimal to mild increases were seen in the epacadostat 600 mg/kg/dose BID alone group (4/6) and in the 9D9 + epacadostat combination groups in 5 out 6 of animals (9D9 + epacadostat 300mg/kg/dose BID) or in all 6 animals (9D9 + epacadostat 600mg/kg/dose BID). As there was only a small difference in liver injury caused by the two epacadostat doses (Fig 3B), only the lower dose was used for the 6-week cohort. After 6 weeks of treatment, minimal to mild increases in inflammation/necrosis were observed in the group treated with anti-CTLA-4 antibody alone (5/6) and minimal to mild increases were observed in the epacadostat 300 mg/kg/dose BID alone group (6/6). Combination treatment revealed minimal to mild increases in inflammation/necrosis (6/6) (Fig 4B). The histopathologic findings were typically characterized by or largely associated with surrounding aggregates of lymphocyte-predominant inflammatory cells. These data demonstrate that after 2 weeks of epacadostat treatment, there was evidence of mild inflammation/necrosis compared to vehicle control, which was potentiated by the addition of anti-CTLA-4 antibody.

GLDH is exclusively localized in the mitochondria of hepatocytes in the liver lobules and released when the cell undergoes necrotic cell death. GLDH can be more sensitive and liver-specific (especially for low-grade ongoing injury) than some of the other common liver biomarkers such as ALT and AST [32]. Consistent with the observation of IDO1 and CTLA-4 inhibition induced hepatocyte necrosis, there was an increase in GLDH serum concentrations in the epacadostat group at 46 days compared to 9D9 only and vehicle control groups. Higher serum GLDH concentrations were observed in the epacadostat + 9D9 combination group as early as day 25 and sustained through day 46 when compared to vehicle and 9D9 groups (Fig 4C). Likely for the reasons explained above, serum concentrations of ALT and AST were not as consistent or as sensitive in correlating with histopathology in the 2-week and 6-week cohorts. Altogether, these data suggest that the combination of inhibiting IDO1, CTLA-4 and PD-1 synergizes to cause liver immune cell infiltration and injury.

### IDO1 and CTLA-4 inhibition-induced liver injury in *PD-1*^*-/-*^ mice is associated with increased numbers of T cells

**Fig 5:**
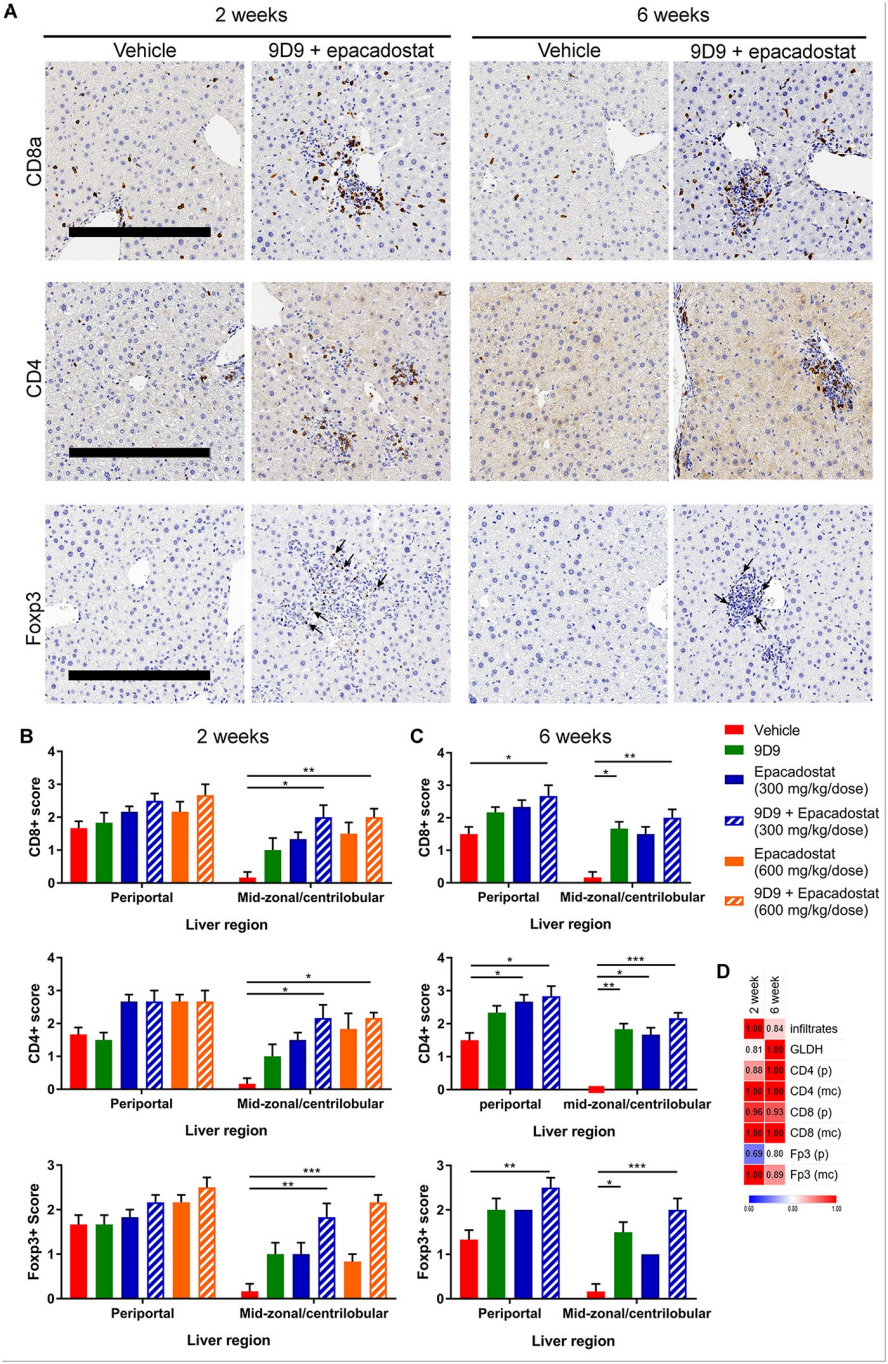
Checkpoint blockade in *PD-1*^*-/-*^ mice increases liver parenchymal T cell inflammation. *PD-1*^*-/-*^ mice were treated with 9D9, lower dose (300 mg/kg BID) epacadostat or in combination for 2 or 6 weeks. (A) Representative images of the mid-zonal/centrilobular region of the liver in the vehicle and combination groups. Semi-quantitative analysis of immunohistochemical detection of CD4, CD8α or Foxp3 at (B) 2 and (C) 6 weeks of treatment. (D) Statistical measure of the strength of association (gamma value) between liver hepatocyte necrosis score with GLDH, immune infiltrates, and immunohistochemistry scores. All data (n = 6 per group) are reported as mean ± SEM. Immune cell scores were analyzed with a Kruskal-Wallis test and Dunn’s multiple comparisons test. Scale bar = 300 μm. *P<0.05, **P<0.01, ***P<0.0001. p: periportal, mc: mid-zonal/centrilobular.

**Fig 6:**
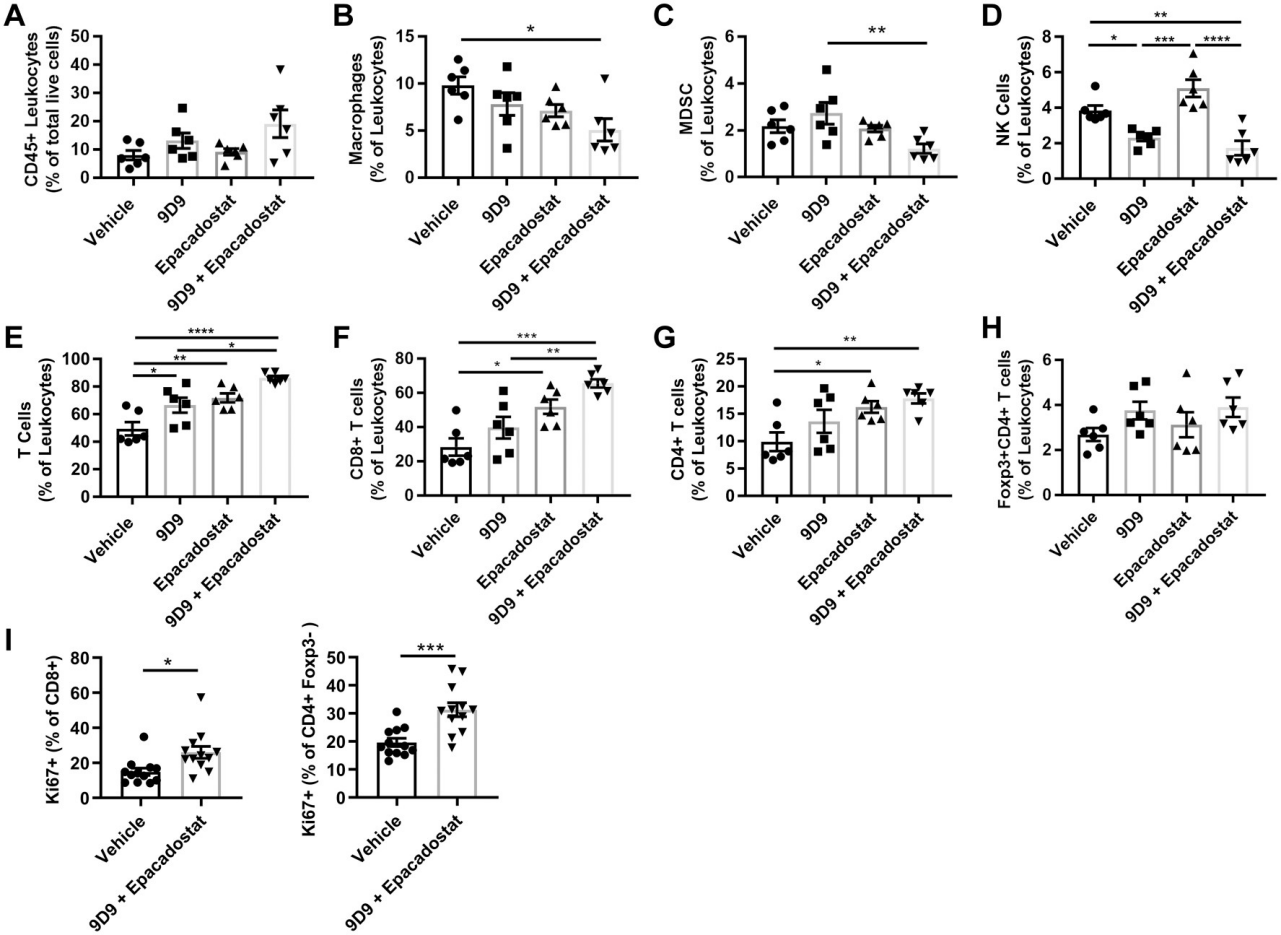
Checkpoint inhibitors increase the number of T cells in the liver of *PD-1*^*-/-*^ mice. Mice were treated with anti-mouse CTLA-4 (9D9), lower dose (300 mg/kg BID) epacadostat or in combination for 6 weeks (A–H) or 3 weeks (I). Single cell suspensions of the whole liver were analyzed for (A) CD45+ cells, (B) CD11b+F4/80+ macrophages, (C) Gr1+CD11b+ myeloid derived suppressor cells (MDSC)s, (D) CD49b+ natural kill (NK) cells, (E) CD3+ T cells, (F) CD3+CD8+ T cells (G) CD3+CD4+ T cells, (H) Foxp3+CD4+ T cells, and (I) Ki67+ T cell subsets by flow cytometry. All data are reported as mean ± SEM. *P<0.05, **P<0.01, ***P<0.001, ****P<0.0001, n = 6 per group, one-way ANOVA with Tukey’s multiple comparisons test (A–H) or n = 12 per group, unpaired t-test (I).

Immune checkpoints inhibit T cell function and proliferation and are crucial to promote self-tolerance in the liver, and as such play a critical role in the modulation of continual antigen stimulation in the natural environment [13]. To investigate further, we hypothesized that IDO1/CTLA-4 blockade-induced liver injury was also associated with increased liver T cells. After 2 weeks of single agent treatment, there was a trend toward increased CD4+, Foxp3+, and CD8+ T cell accumulation in the mid-zonal/centrilobular areas of the liver as demonstrated by immunohistochemistry analysis. There was approximately a two-fold increase in CD4+, Foxp3+ and CD8+ cells in mice co-administered epacadostat + 9D9 when compared to vehicle control data (Fig 5A and 5B). Since the increase in T cells was similar at both doses of epacadostat at 2 weeks, a separate cohort was analyzed at 6 weeks treated with the lower dose (300 mg/kg BID) only. At 6 weeks, there was an increase in T cells with epacadostat + 9D9 treatment in the periportal as well as the mid-zonal/centrilobular areas of the liver (Fig 5A and 5C). These results suggest early T cell infiltration at the areas of inflammation/necrosis in the mid-zonal/centrilobular regions, which in some cases progressed to a more widespread liver inflammation at later time points. The severity of T cell accumulation was highly correlated with inflammation/necrosis in both regions of the liver at 2 and 6 weeks (Fig 5D). A more detailed flow cytometry analysis supported and was consistent with the immunohistochemistry results. The epacadostat + 9D9 combination treatment increased the percentages of total T cells, CD4+ and CD8+ T cells, and reduced the percentages of macrophages and natural killer cells in the liver (Fig 6). The combination treatment induced Ki67+ in CD4 and CD8+ T cells, suggesting the increase in T cells was due in part to proliferation (Fig 6I). The gating strategy for these immune cell populations is shown in S1 Fig. To determine if the quantity of immune cell populations was associated with inflammation/necrosis in the liver, a linear regression analysis was performed. As shown in Fig 6, T cell populations were positively correlated with necrosis severity, while macrophages were negatively correlated with necrosis. There was no association of Tregs, myeloid derived suppressor cells, or natural killer cells with necrosis.

**Fig 7:**
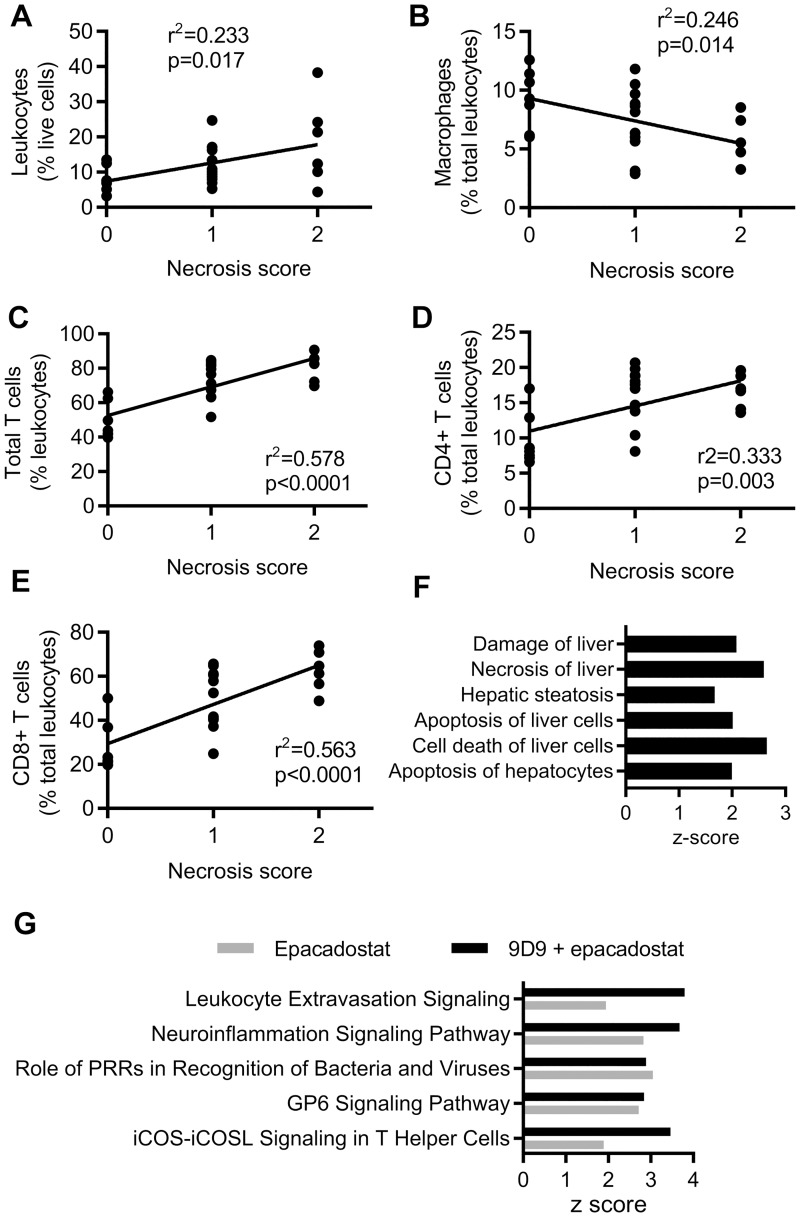
T cell infiltration of the liver positively associates with necrosis. Mice were treated with anti-mouse CTLA-4 (9D9), 300 mg/kg BID epacadostat or in combination for 6 weeks (A-E) or with 600 mg/kg BID epacadostat or in combination for 2 weeks (F-G). Linear regression analysis of (A) leukocytes (CD45+ cells), (B) macrophages, (C) total T cells and (D) CD4+ and (E) CD8+ T cells as defined in Fig 6 with single cell necrosis. (F) The top activated (> 1.5 z-score) toxicity pathways significantly modulated by 9D9 + epacadostat treatment compared with vehicle control, sorted by p-value and (G) the top five significantly regulated canonical pathways relative to vehicle identified by ingenuity pathway analysis (sorted by combination vs vehicle ascending p-values). There were no significant changes, and thus no activation of these canonical pathways, in the 9D9 vs vehicle group. PRR: pattern recognition receptor, ICOS: inducible T-cell costimulator ICOSL: inducible T-cell costimulatory ligand.

To understand the molecular mechanisms underlying the CPI induced liver injury, transcriptomic analysis was performed on liver tissues at 2 weeks. This time-point was chosen to detect early changes induced by CPI that are less likely to be confounded by downstream responses to progressive hepatocyte necrosis and tissue damage. Pathway analysis identified increased expression of genes associated with hepatocyte necrosis and apoptosis pathways (Fig 7F) and several immune signatures (Fig 7G). Notably, leukocyte trafficking and CD4 T cell function was synergistically activated by the combination treatment. Innate pathways and platelet activation were induced by epacadostat but not potentiated with anti-CTLA-4. Additionally, the checkpoint inhibitor combination increased activation of neutrophil, monocyte, macrophage and B cell pathways in PD-1 knockout mice; suggesting a role for other inflammatory cells in CPI-mediated liver injury (S1 Table). Altogether, these data suggest that the combination of CTLA-4 blockade and IDO1 inhibition enhances T cell infiltration, activation and liver injury.

## Discussion

In this study, IDO1 or CTLA-4 blockade in C57BL/6 mice induced liver injury, which was exacerbated when these checkpoint inhibitors were used in combination. These effects were more substantial when these CPIs were given to PD-1 deficient mice. Therefore, these mouse models were able to recapitulate the clinically observed enhanced liver toxicity during treatment with various combinations of CTLA-4, PD-1 and IDO1 inhibitors. Treatment of *PD-1*^*-/-*^ mice with anti-CTLA-4 or an IDO1 inhibitor induced liver lobule inflammation, consisting of CD4+ and CD8+ T cells, into all zones of the liver parenchyma, with necrosis predominating in mid-zonal and centrilobular areas. The degree of liver T cell inflammation was positively correlated with the extent of hepatocyte necrosis, suggesting that hepatocyte cell death was mediated by these T cells. This was also supported by the observation of a common phenomenon of small clusters of necrotic cells surrounded by a ring-like cluster of lymphocytes. Transcriptome analysis of the liver demonstrated that inhibition of IDO1 and CTLA-4 in *PD-1*^*-/-*^ mice induced hepatocyte cell death pathways and a strong immune signature including leukocyte trafficking and activation of T cells.

In patients treated with ipilimumab and nivolumab, case reports displayed a somewhat broader but frequently similar array of pathological changes in the liver that may be reflective of greater age and lesion stage variation. T cell infiltration was identified as a consistent feature of immune related adverse events associated with CPIs [16, 33, 34]. Focal necrosis throughout the liver parenchyma with predominantly CD8+ T cell infiltration was also commonly observed in patients treated with CPIs [35, 36]. These human cases characterized in the literature are likely representative of the more severe cases of immune-mediated hepatotoxicities associated with CPI treatment, and therefore it is not surprising that the histopathologic images reported from those lesions are most similar to a few of the most severe cases we identified in our mouse model. This includes the coalescence of smaller inflammatory and necrotic foci into larger regions of necrosis, at which time structural impairment leads to further nonspecific histopathologic features of injury.

There are numerous independent and occasionally synergistic causes identified for general drug induced liver injury (DILI). Liver injury specifically mediated by IO agents may be caused by distinct mechanisms, and therefore modeled separately, from chemically-induced, hepatocyte-intrinsic cell damage and death. In the latter, hepatocyte cell death is typically the primary initiator of the inflammatory response [37]. Regarding immune-mediated hepatotoxicity, there is a predominance of infiltrating immune cells associated with variable amounts of hepatocellular necrosis. It should be noted however that some of the histopathologic features attributed to immune CPI toxicities were similar and some were different when compared to cases of autoimmune hepatitis or idiosyncratic DILI in humans [36]. Here we showed that CPI induced liver injury is primarily mediated by T cell immunity with a characteristic histological finding of necrotic hepatocytes surrounded by ring-like aggregates of T cell-predominant mononuclear cells. Pathway analysis revealed that the inhibition of IDO1 and CTLA-4 activated both hepatic necrosis and apoptosis pathways in *PD-1*^*-/-*^ mice. Oxidative stress pathways were also activated with inhibition of IDO1 and CTLA-4 and may contribute to the mechanism of hepatocyte cell death. At the physiological level, the liver experiences a heavy antigenic load from filtering environmental agents derived from the gastrointestinal tract, excretion of toxic waste substances, and uptake of blood-borne pathogens. Thus, the liver has multiple mechanisms to promote immune tolerance including the CTLA-4 and CD80/86, PD-1 and PD-L1/PD-L2, and IDO1 pathways. It can be presumed that some genetic variance may influence different inherent susceptibilities between individuals. Within this context, inhibition of these separate pathways may result in a synergistic loss of tolerance and immune-mediated hepatocyte cell death. Furthermore, some CPIs can also induce the expression of other checkpoint molecules, which may explain the synergistic effect of combining CPIs. Activation of 41BB alone and in combination with anti-PD-1 can also induce liver injury [38]. OX40 ligand and CD30 ligand blockade have been shown to attenuate the anti-CTLA-4 and anti-PD-1 combination-induced liver toxicity [23]. Cancer immunotherapeutic-induced hepatotoxicity is commonly characterized by focal necrosis with surrounding immune cells, suggesting a mechanism of immune-mediated hepatocyte killing.

The hepatocyte cell death observed with CPIs is likely a necroinflammatory process. The observation of individual or small clusters of necrotic hepatocytes suggests a mechanism of immune mediated cell death, in contrast to the more zonal and/or focally extensive patterns of necrosis often identified as consequential to ischemia or direct cytotoxicity. Even in cases of single cell or small foci of necrosis associated with primary toxicities, rings of surrounding mononuclear cells are generally not a predominant feature. Indeed, leukocyte trafficking pathways were synergistically activated by IDO1 and CTLA-4 inhibition in *PD-1*^*-/-*^ livers. Activated lymphocytes have been shown to mediate hepatocyte and cancer cell death through expression of death ligands, granule exocytosis, or by production of cytokines. Tumor necrosis factor, first apoptosis signal receptor, and tumor necrosis factor -related apoptosis-inducing ligand have been shown to trigger cell death in hepatocytes [39–41]. CD8+ T cells can induce hepatocyte cell death via the first apoptosis signal receptor and cytolytic effector molecules [41]. CD4+ T cells likely support CD8+ T cell and macrophage function by production of cytokines. In *PD-1*^*-/-*^ mice, inhibition of IDO1 and CTLA-4 activated the ICOS-ICOSL signaling pathway in CD4 T cells. CTLA-4 antagonism has been shown to activate this pathway and promote interferon gamma production, an important cytokine that can mediate cell death, by CD4+ T cells in humans [42]. In the human liver, IO agent-induced liver toxicity is associated with increased numbers of CD4+ T, CD8+ T and CD20+ B lymphocytes [36].

Beyond T lymphocytes, other inflammatory cells may also play a role in CPI-induced liver injury. Antigen presenting cells such as Kupffer cells and DCs can express IDO1 and PDL1 to inhibit liver CD8 T cells and promote Treg function [43]. Thus, CPI treatment may enable liver antigen presenting cells to activate CD8 T cells and suppress Tregs, thereby promoting liver injury. There is evidence for a role of macrophages as Fcγ Receptor-mediated Phagocytosis in Macrophages and Monocytes, CCR5 Signaling in Macrophages, Production of Nitric Oxide and Reactive Oxygen Species in Macrophages, and GM-CSF signaling pathways are activated with CPI treatment in the transcriptome analysis. Other lymphocytes have shown to play a role in DILI [44]. In this study, B cell functional pathways B Cell Receptor Signaling, PI3K Signaling in B Lymphocytes and FcγRIIB Signaling in B Lymphocytes, were activated with CPI treatment. CD4 T cells can activate B cells to produce antibodies that mediate cellular cytotoxicity [44]. NK and NKT cells may produce cytokines that modulate the microenvironment or promote cell death through their cytolytic function [45]. The exact role of these inflammatory cells in our CPI-induced liver injury mouse model have yet to be determined.

Treatment of *PD-1*^*-/-*^ mice with checkpoint inhibitors resulted in an elevation of GLDH with greater sensitivity and correlation with histopathology than other biomarkers of liver injury such as ALT and AST. As previously stated, this is likely consequential to the low-grade, ongoing nature of the inflammatory process in these mice which is reflective of the spectrum of severity that can occur with immune-mediated mechanisms of hepatocyte cell death due to normal individual variation. GLDH has been recognized as one of the most useful liver injury biomarkers in mice with greater sensitivity and liver specificity than other biomarkers such as ALT and AST. Other models of hepatic injury have confirmed that GLDH can have higher fold increases and persist longer than ALT and even occur when plasma ALT is not elevated [32]. In CPI induced liver injury, our findings and internal experience suggest that GLDH may be a more sensitive marker than ALT and/or AST.

CPIs are the cornerstones for future combination therapies with IO and non-IO drugs and have the potential to further improve clinical efficacy. These combinations may pose a significantly increased risk of augmented immune-related adverse events including but not limited to hepatitis, pneumonitis and myocarditis, in some cases with potentially severe outcomes. In the current study, a mouse model was used to investigate the immune mechanisms of one of the more commonly seen immune-related adverse events, hepatitis, in the setting of combinatorial checkpoint blockade. This model could be of significant value in further and deeper characterization and mechanistic investigation of immune-mediated liver toxicities associated with various drug combinations, thus providing circular guidance and support for clinical anti-cancer drug combination regimens that contain CPIs.

## Supporting information

S1 Fig Flow cytometry gating strategy for leukocyte populations in the liver. Live (7AAD-) cells were gated for CD45+ then for NK cells (CD49b+), macrophages (CD11b+ F4/80+), MDSCs (Gr1+ CD11b+) or T cells (CD3+). T cells were further gated for CD4+, or CD8+, or Tregs (CD4+Foxp3+).
(DOCX)

S1 Table Canonical pathways identified by ingenuity pathway analysis. Mice were treated with anti-mouse CTLA-4 (9D9), 600 mg/kg BID epacadostat or in combination for 2 weeks. The complete list of significantly regulated (p < 0.05) canonical pathways identified by ingenuity pathway analysis (sorted by combination vs vehicle ascending p-values).
(XLS)
