# Supplementary material for: Inhibition of immune checkpoints PD-1, CTLA-4, and IDO1 coordinately induces immune-mediated liver injury in mice
Source: PLoS One. 2019 May 21;14(5):e0217276. doi: 10.1371/journal.pone.0217276 (PMC6528985; doi:10.1371/journal.pone.0217276)
Supplement: S1 Fig — Live (7AAD-) cells were gated for CD45+ then for NK cells (CD49b+), macrophages (CD11b+ F4/80+), MDSCs (Gr1+ CD11b+) or T cells (CD3+). T cells were further gated for CD4+, or CD8+, or Tregs (CD4+Foxp3+). (DOCX) [file pone.0217276.s001.docx]

Supplemental material

Inhibition of immune checkpoints PD-1, CTLA-4, and IDO1 coordinately induces immune-mediated liver injury in mice


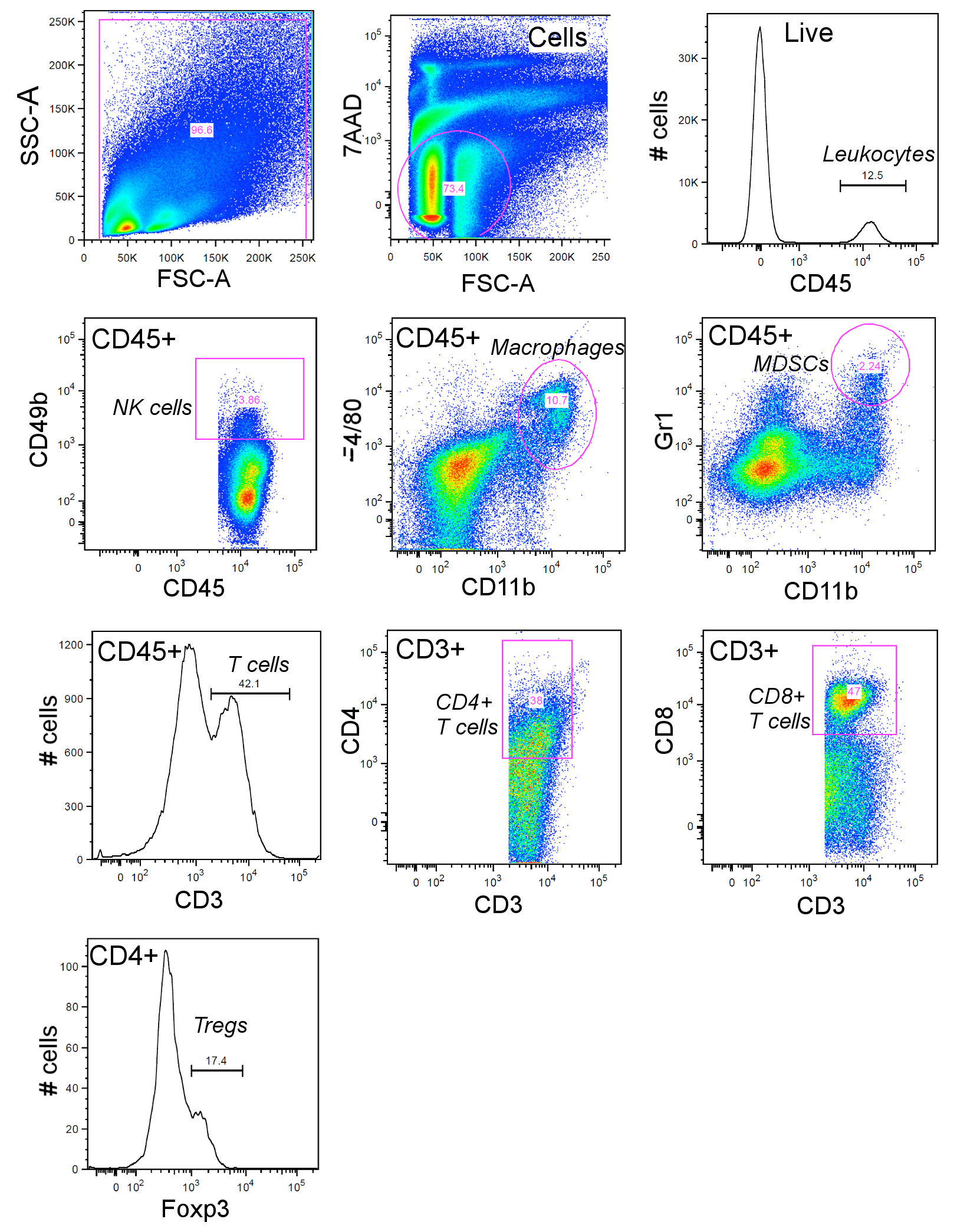


Supplemental figure I. Flow cytometry gating strategy for leukocyte populations in the liver. Live (7AAD-) cells were gated for CD45+ then for NK cells (CD49b+), macrophages (CD11b+ F4/80+), MDSCs (Gr1+ CD11b+) or T cells (CD3+). T cells were further gated for CD4+, or CD8+, or Tregs (CD4+Foxp3+).
